# An Update on *in Vivo* Imaging of Extracellular Vesicles as Drug Delivery Vehicles

**DOI:** 10.3389/fphar.2018.00169

**Published:** 2018-02-28

**Authors:** Prakash Gangadaran, Chae Moon Hong, Byeong-Cheol Ahn

**Affiliations:** Department of Nuclear Medicine, School of Medicine, Kyungpook National University and Hospital, Daegu, South Korea

**Keywords:** extracellular vesicles, drug delivery vehicles, molecular imaging, *in vivo* distribution, labeling

## Abstract

Extracellular vesicles (EVs) are currently being considered as promising drug delivery vehicles. EVs are naturally occurring vesicles that exhibit many characteristics favorable to serve as drug delivery vehicles. In addition, EVs have inherent properties for treatment of cancers and other diseases. For research and clinical translation of use of EVs as drug delivery vehicles, *in vivo* tracking of EVs is essential. The latest molecular imaging techniques enable the tracking of EVs in living animals. However, each molecular imaging technique has its certain advantages and limitations for the *in vivo* imaging of EVs; therefore, understanding the molecular imaging techniques is essential to select the most appropriate imaging technology to achieve the desired imaging goal. In this review, we summarize the characteristics of EVs as drug delivery vehicles and the molecular imaging techniques used in visualizing and monitoring EVs in *in vivo* environments. Furthermore, we provide a perceptual vision of EVs as drug delivery vehicles and *in vivo* monitoring of EVs using molecular imaging technologies.

## Introduction

Extracellular vesicles (EVs) are naturally occurring nanovesicles released by different types of cells, including reticulocytes (Johnstone et al., 1991), platelets (Brisson et al., 2017), mesenchymal stem cells (Rajendran et al., 2017), T cells (Karlsson et al., 2001; Ludwig et al., 2017), B lymphocytes (Raposo et al., 1996), NK cells (Shoae-Hassani et al., 2017; Zhu et al., 2017), dendritic cells (DCs) (Lu et al., 2017), and some tumor cells (Aharon et al., 2017; Baumgart et al., 2017; Schillaci et al., 2017); these nanovesicles can be detected in human biological fluids (Cappello et al., 2017). EVs include exosomes (small membranous vesicles) and microvesicles (large membranous vesicles) shed by cells (Di Rocco et al., 2016; Gangadaran et al., 2017a). In this article, we use the term “EVs” to refer to both exosomes and microvesicles.

EVs have recently gained attention as mediators of cellular communication (Srivastava et al., 2015). Several studies suggest that EVs are not merely secreted to provide a degradation route for unwanted biological materials (Johnstone et al., 1991) but are equipped to withstand lysis by the complement system to perform vital extracellular functions (Clayton et al., 2003).

EVs can carry various biological constituents such as lipids, proteins, and nucleic acids (Yáñez-Mó et al., 2015). These molecules are packed into EVs from the host cell cytoplasm through endosomal sorting complexes (Gangadaran et al., 2017a). These molecules and other contents of the EVs act on target cells. EVs from several cells induce apoptosis in tumors and induce anti-tumor immune responses (Filipazzi et al., 2012; Kalimuthu et al., 2016; Zhu et al., 2017). Moreover, serum-derived EVs and mesenchymal stem cell (MSC)-derived EVs increase the angiogenic activities of endothelial cells (Cavallari et al., 2017; Rajendran et al., 2017).

Accumulating studies suggest the importance of EVs in long distance cell-cell communication because the secreted EVs can enter the circulation and pass through additional biological barriers (Jiang and Gao, 2017; Sarko and McKinney, 2017). EVs are used as carriers for anticancer drugs (Agrawal et al., 2017), miRNA (Yang et al., 2017), and siRNA (Vader et al., 2017), and are now considered as promising drug transporters.

To utilize EVs as drug delivery vehicles, *in vivo* tracking of EVs to target organs is warranted. Non-invasive imaging modalities can provide accurate *in vivo* distribution and kinetics of the EVs and provide better understanding of the *in vivo* therapeutic effects of EVs. Recent advances in molecular imaging modalities allow us to well-recognize both cellular and subcellular biological processes within living subjects (Lee et al., 2016; Li et al., 2016). However, labeling procedures are necessary for accurate *in vivo* visualization of certain biomaterials in an animal model. EVs can be directly labeled using various agents such as, lipophilic tracer dyes (Ohno et al., 2013; Grange et al., 2014), radionuclides (Hwang et al., 2015), or magnetic particles (Piffoux et al., 2017) and indirectly labeled with a reporter gene (luciferase or fluoresce) by transducing the gene in originating cells (Koumangoye et al., 2011; Lai et al., 2014; Hoshino et al., 2015; Gangadaran et al., 2017b).

In this paper, we will discuss EVs as drug delivery vehicles, labeling techniques used for molecular imaging of EVs to evaluate their biodistribution and lesion targeting, merits and demerits of the labeling methods, and EV-based targeted drug delivery in diseases. Furthermore, we review the technology developments and strategies that led to the current state-of-the-art techniques used for EV visualization with specific *in vitro* and *in vivo* examples.

## Biogenesis of EVs

**Figure 1:**
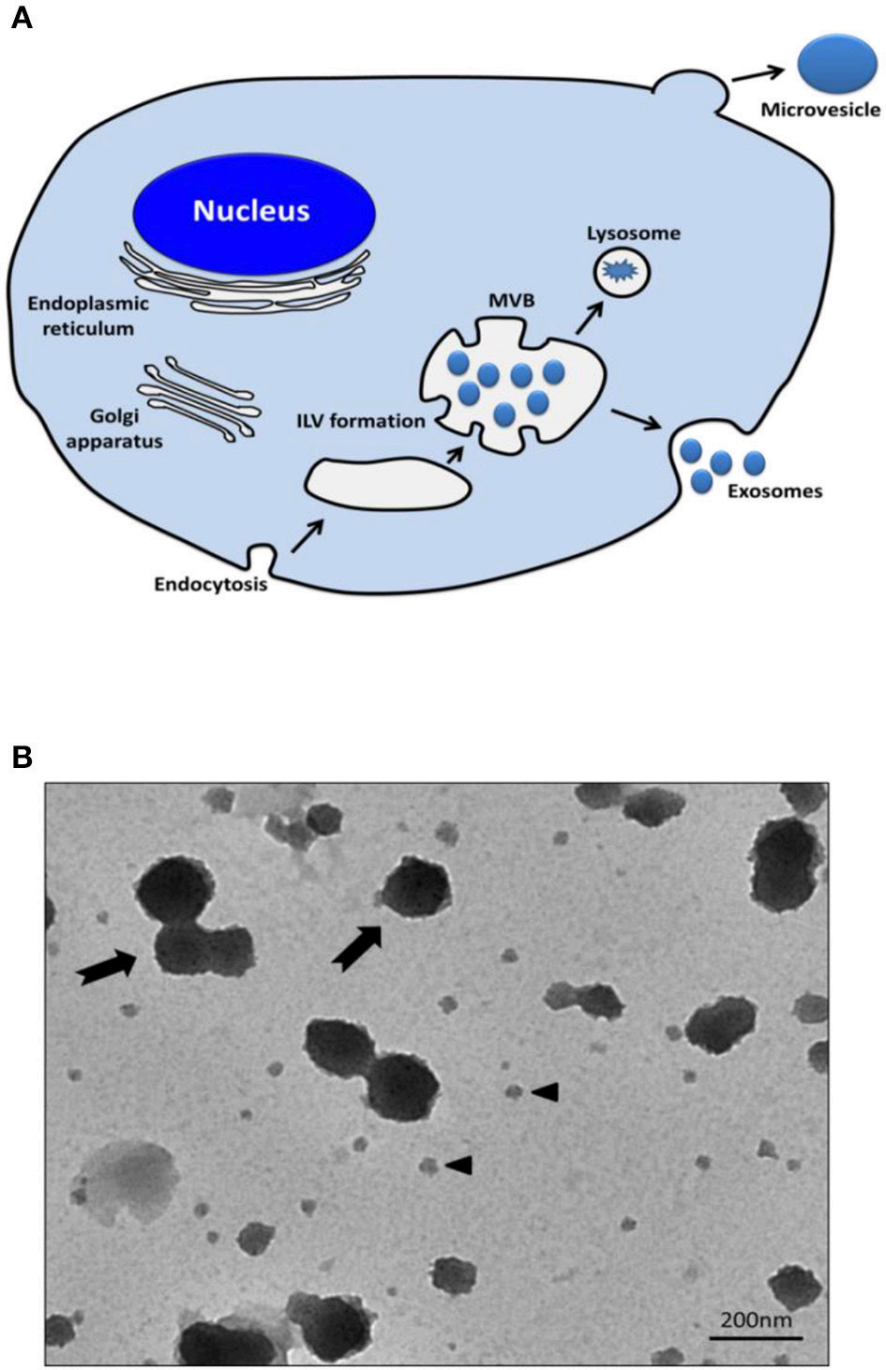
Release of exosomes and microvesicles. **(A)** Exosomes are represented by small vesicles of different sizes, released from MVB whereas microvesicles bud directly from the plasma membrane. **(B)** TEM images showing the typical structure of exosomes (black arrow head) and microvesicles (black arrows). MVB, Multi-vesicular bodies; ILV, intraluminal vesicles; TEM, transmission electron microscopy.

EVs are nanosized membrane vesicles released by cells into the extracellular space and are found in various body fluids such as blood, urine, and central nervous system fluids. EVs are classified into exosomes and microvesicles; exosomes (50–200 nm) are membrane vesicles released by multi-vesicular bodies, whereas microvesicles (50–1,000 nm) are released from the cell membrane via the budding process and they are larger than exosomes (Figures 1A,B; Gangadaran et al., 2017a).

Several exosome production pathways have been identified. The endosomal sorting complex required for transport (ESCRT) and related proteins such as programmed cell death 6 interacting protein (PDCD6IP; also known as ALIX) and tumor susceptibility gene 101 (TSG101) protein are associated with the cargo sorting of exosomes. Moreover, ESCRT-independent mechanisms, such as ceramide-related pathway, also operate to generate exosomes of certain biochemical compositions (Trajkovic et al., 2008; Bobrie et al., 2011). Then, the exosomes are released from the cells by membrane fusion of the exosome-containing multivesicular body with the cell membrane. In contrast, microvesicles are formed by the outward budding and fission of the cell membrane. These processes are controlled by membrane lipid microdomains and regulatory proteins such as ADP-ribosylation factor 6 (Muralidharan-Chari et al., 2009). The membrane composition of microvesicles reflects that of the parent cell more closely than that of exosomes because of their different biogenesis mechanisms. Both exosomes and microvesicles contain various biological materials, but microvesicles are a relatively heterogenous population of vesicles, compared to exosomes (Théry et al., 2009; EL Andaloussi et al., 2013).

Recently, extracellular vesicle mimetics (EVMs), also called artificial nanovesicles, are being considered as new drug delivery vehicles. Large quantities of cells and culture medium are needed to obtain the desirable EVs; however, EVM production is less time-consuming and laborious (Kim et al., 2017b). EVMs were prepared by breaking down the cells through serial extrusion using nanosized filters with diminishing pore sizes and were isolated using density gradient ultracentrifugation (Jang et al., 2013).

EVs can carry various biological materials like lipids, proteins, mRNA, miRNA, and extra-chromosomal DNA. A recent study revealed that EVs contain 4,563 proteins, 194 lipids, 1,639 mRNAs, and 764 miRNAs (Mathivanan et al., 2012). The protein content of EVs is related to their originating cell type and their biogenesis. Recent proteomic study using EVs originated from DCs showed that exosomes are additionally characterized by the presence of the tetraspanins (CD9, CD63, and CD81), the ESCRT protein TSG101, and syntenin. In contrast, a number of factors such as class II major histocompatibility complex (MHCII), flotillin, or heat shock 70 kDa proteins were found in both exosomes and microvesicles (Kowal et al., 2016). EVM showed similar protein markers, such as CD9, ALIX, and TSG101. Membrane lipids of EVM were more similar to those of exosomes than those of their parent cells (Goh et al., 2017).

Until 1973, EVs were considered as a garbage materials released by the cells (Nolte-‘t Hoen et al., 2016). However, recent studies demonstrated that EVs are vital cell-to-cell communication messengers between distant cells and that EVs can attach to a cell surface or enter into recipient cells (Escrevente et al., 2011). EVs serve as a carrier and can transfer information from the parent cells to their target cells (Kalimuthu et al., 2016; Gangadaran et al., 2017c; Zhu et al., 2017).

**Figure 2:**
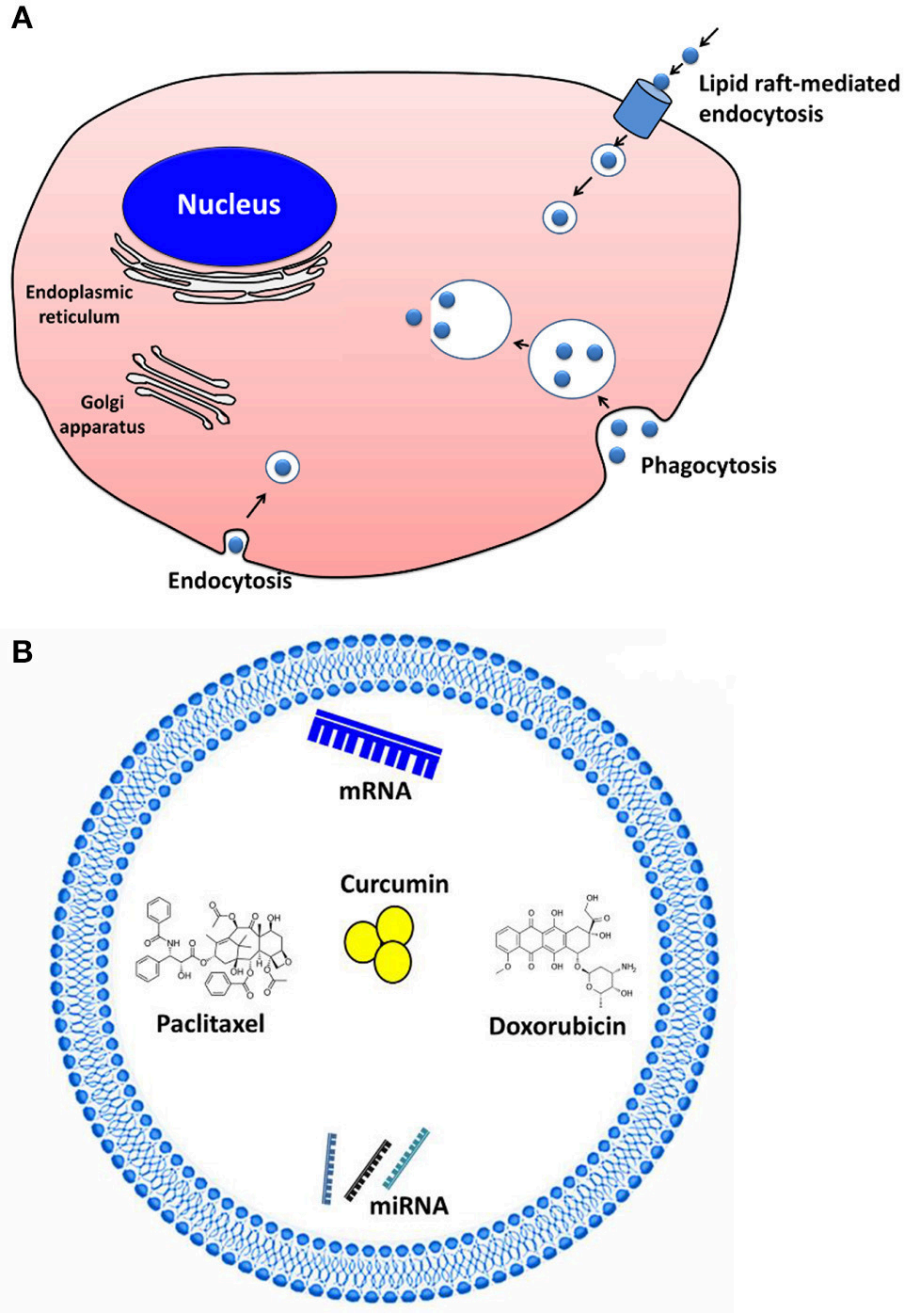
Extracellular vesicles (EVs) as drug delivery vehicles and their communication**. (A)** Cells internalize EVs by various endocytic pathways, including clathrin-dependent endocytosis, caveolin-mediated uptake, micropinocytosis, phagocytosis, and lipid raft–mediated internalization. **(B)** The therapeutic cargo can contain different types of interfering RNAs, mRNA, or even therapeutics (e.g., paclitaxel, doxorubicin, curcumin) to treat cancer and inflammatory diseases.

Cells internalize EVs by various endocytic pathways, including clathrin-dependent endocytosis (Escrevente et al., 2011), caveolin-mediated uptake, micropinocytosis (Fitzner et al., 2011), phagocytosis (Hemler, 2003), and lipid raft-mediated internalization (Svensson et al., 2013; Figure 2A). EVs may enter the target cell via more than one route. The uptake mechanism may depend on the proteins and glycoproteins present on the surface of both EVs and the target cell (Mulcahy et al., 2014).

## EVs as drug delivery vehicles

Recently, the most studied drug delivery platform are liposomes and polymeric nanoparticles. Liposomes are tiny vesicles composed of fatty acid-containing bilayers surrounding an aqueous core, and they have established their roles as carriers for therapeutic drugs (Sercombe et al., 2015). Polymeric nanoparticles are drug delivery platform that help in the encapsulation, entrapment, or attaching the drug molecules (Kamaly et al., 2016). Both liposome and polymeric nanoparticles have been used to deliver various drug molecules, such as anti-cancer drugs (Hofheinz et al., 2005; Masood, 2016). However, synthesis of non-toxic liposomes with high stability, ability to circulate for a long time, and with the ability to evade the host immune system, remains a concern (Sercombe et al., 2015). Polymeric nanoparticles showed a better stability than liposomal systems but their biocompatibility needs to be evaluated (Li et al., 2015).

Several recent reports have shown the advantages of using EVs for drug delivery: (i) EVs are small and can penetrate into deep tissues (Gangadaran et al., 2017b); (ii) they possesses negative zeta potential for long circulation (Malhotra et al., 2016); (iii) EVs have membrane structure similar to that of cells (Hood and Wickline, 2012); (iv) EVs exhibit an increased ability to escape degradation (Luan et al., 2017); (v) EVs can evade the immune system (Anderson et al., 2016; Kamerkar et al., 2017). In addition, few human clinical trials performed using EVs from DCs for cancer therapy, reported positive results with respect to the feasibility and safety of EVs (Escudier et al., 2005; Morse et al., 2005; Besse et al., 2016). Overall, EVs are clinically applicable, excellent, natural carriers mainly because of their inherent biocompatibility.

However, to qualify as drug delivery vehicles, EVs should be able to carry a substantial amount of therapeutics. A variety of cargos have now been shown to exhibit therapeutic effect after EV-based delivery (Sun et al., 2010; Banizs et al., 2014; Pascucci et al., 2014; Tian et al., 2014; Figure 2B). EVs were reported to deliver the anti-inflammatory agent curcumin to activated myeloid cells in a mouse model. The curcumin delivered by EVs was more stable *in vivo* and more highly concentrated in the mouse blood (Sun et al., 2010). Other studies reported that EVs can be used for the delivery of pharmaceutical drugs like paclitaxel and doxorubicin to inhibit tumor growth both *in vitro* and in animal models (Pascucci et al., 2014; Tian et al., 2014). Furthermore, EVs were suggested to be useful as nanocarriers for exogenous siRNA to control gene expression in the recipient cells (Wahlgren et al., 2012; Banizs et al., 2014). Using EVs, an antitumor miRNA (let-7a miRNA) was delivered to EGFR-expressing xenograft breast cancer cells in mice; this delivery system efficiently inhibited the tumor growth (Ohno et al., 2013). In mouse models of pancreatic cancer, exosomes containing siRNA or shRNA specific to oncogenic KRAS showed increased therapeutic effects, compare to the controls including liposomes containing siRNA or shRNA (Kamerkar et al., 2017).

Although the use of EVs as systems to deliver therapeutic materials has been widely studied (Table 1), the effectiveness of EV-based therapy depends on the targetability of EVs to tumor or another desired cell *in vivo*. Non-invasive imaging modalities might provide clear view on the *in vivo* distribution of EVs and provide accurate targetability of EVs to the desired cell/tissue and would be useful in the development of EVs as drug delivery vehicles.

**Table 1:** Examples of studies that used extracellular vesicles as drugs delivery vehicles.

**Drug**		**Loading method**	**Type of EVs**	**Outcome**	**References**
Small molecules	Curcumin	Incubation	Exosome	Increased the anti-inflammatory activity of Curcumin	Sun et al., 2010
	Cucurbitacin-I and curcumin	Incubation	Exosome	Increased neuroprotective effects	Zhuang et al., 2011
	Catalase	Incubation/Sonication/Extrusion/Freeze/thaw	Exosome	Increased neuroprotective effects	Haney et al., 2015
Anti-cancer drugs	Paclitaxel or Doxorubicin	Incubation	Exosome	Delivered anticancer drug to the brain	Yang et al., 2015
	Doxorubicin	Electroporation	Engineered exosome	Inhibited tumor growth	Tian et al., 2014
	Paclitaxel	Incubation/Electroporation/Extrusion	Exosome	Overcome MDR in cancer cells	Kim et al., 2016
	Paclitaxel	Incubation	Microvesicles and exosome	Cancer cell-derived EVs increased cytotoxicity	Saari et al., 2015
siRNA	BACE1	Electroporation	Exosome	Enables specific gene knockdown	Alvarez-Erviti et al., 2011
	MAPK1	Electroporation	Plasma exosomes	Transported exogenous siRNA to human blood cells	Wahlgren et al., 2012
	^#^RAD51 and RAD52	Chemical treatment and electroporation	Exosomes	siRNA against RAD51 was functional and resulted in cell death of recipient cancer cells.	Shtam et al., 2013
miRNA mimics/inhibitor	miR-155-mimics/inhibitor	Electroporation	Exosome	Changed the biological response in hepatocytes and macrophages.	Momen-Heravi et al., 2014
	miR-15a mimic/inhibitor	Transfection	Exosome	Enabled highly efficient overexpression or deletion of the designated miRNAs	Zhang et al., 2017

siRNA, silencing RNA; miRNA, microRNA; BACE1, Beta-secretase 1; MAPK1, Mitogen-activated protein kinase 1;

# *RAD51/52 is involved in DNA repair of double-strand breaks and homologous recombination*.

## Investigating EVs by molecular imaging

**Figure 3:**
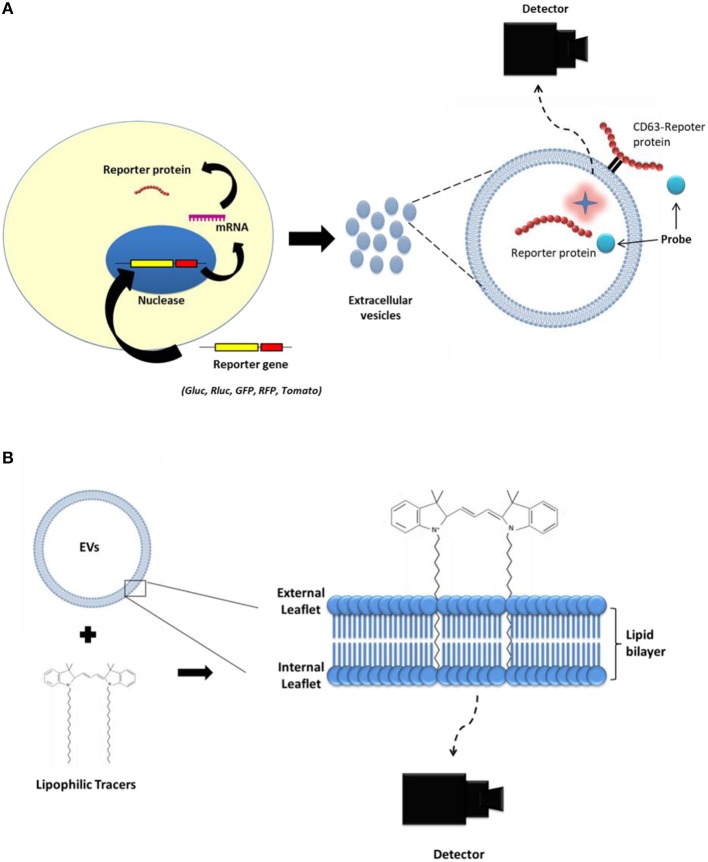
Strategies to label extracellular vesicles (EVs) for optical imaging. **(A)** First, reporter genes (*Gluc, Rluc, GFP, RFP, tdTomato*) are transduced into the parent cell line. Then, EVs produced from the parent cells expressing the reporter protein carry the reporter protein inside their lumen or on their membrane. **(B)** Lipophilic imaging agents (such as, DiD and DiR) could bind to the membrane of the EVs.

**Figure 4:**
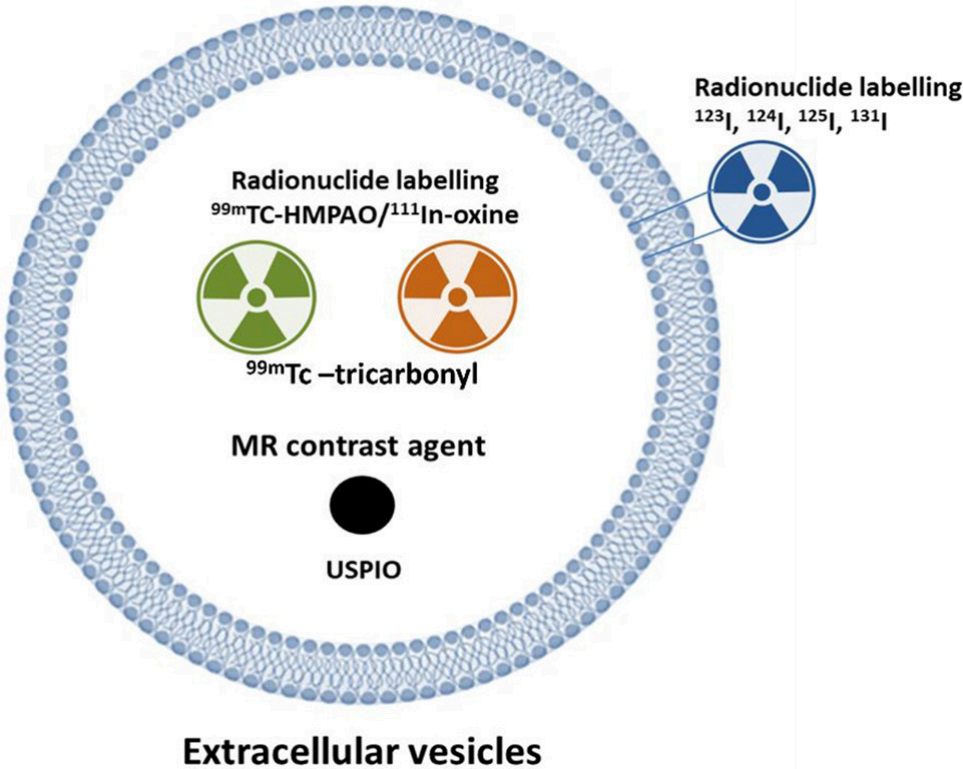
Strategy to label extracellular vesicles (EVs) for nuclear or MR imaging. Complex of ^111^In-oxine and ^99m^Tc-HMPAO are lipophilic and penetrate the membrane of cells or extracellular vesicles (EVs). Inside the EVs, ^111^In-oxine attaches to cytoplasmic components (such as lactoferrin). ^99m^Tc-HMPAO reacts with glutathione inside the EVs and it is converted to hydrophilic form. ^99m^Tc**-**tricarbonyl binds to amino acids (such as histidine, methionine, and cysteine) of the EV membrane. EVs expressing lactadherin-streptavidin fusion protein on the membrane bind with the radioiodine-labeled biotin. USPIO was loaded to EVs using electroporation or incubation. HMPAO: hexamethylpropyleneamineoxime; USPIO: ultra-small super paramagnetic iron oxide.

Several molecular imaging strategies [optical, nuclear, and magnetic resonance imaging (MRI); Figures 3, 4] have been employed for *in vitro, in vivo*, and *ex vivo* tracking of EVs to determine their biodistribution in animal models and targeting of certain EVs via various delivery routes in small animal models for different diseases (Table 2). However, EVs derived from tumors or other cells influence the tumor itself as well as the tumor microenvironment, and the EVs are able to accelerate or inhibit growth and metastasis of the tumor (Hoshino et al., 2015; Liu and Cao, 2016; Schillaci et al., 2017; Wang et al., 2017). Therefore, a better understanding of EV biodistribution after administration is warranted for the safe and effective clinical application of EV-based therapies for various diseases (Lener et al., 2015).

**Table 2:** Tracking of extracellular vesicle biodistribution using molecular imaging strategies.

**Imaging technique**	**Labeling strategy**	**Labeling molecule**	**EV source**	**Administration route**	**Subject**	**Clinical translation**	**References**
FLI	Direct	DiD	Breast cancer cells	IV	Mice	X	Wen et al., 2016
		DiR	HEK293T, C2C12-, B16F10-, and DC	IV, IP, IM	Mice	X	Wiklander et al., 2015
			HEK293	IV	Mice	X	Watson et al., 2016
			CAL62	IV	Mice	X	Gangadaran et al., 2017b
BLI	Indirect	Gluc	B16BL6	IV	Mice	X	Imai et al., 2015
			B16BL6	IV	Mice	X	Takahashi et al., 2013
			HEK293T	IV	Mice	X	Lai et al., 2014
			B16BL6, C2C12, NIH3T3, MAEC, and RAW264.7	IV	Mice	X	Charoenviriyakul et al., 2017
		Rluc	CAL62, MDA-MB-231	IV	Mice	X	Gangadaran et al., 2017b
NI	Direct	^99m^Tc-HMPAO	Raw 264.7 and HB1.F3 cells	IV	Mice	O	Hwang et al., 2015
		^99m^Tc-tricarbonyl	RBC	IV	Mice	O	Varga et al., 2016
		^125^I-biotin	B16BL6	IV	Mice	X	Morishita et al., 2015

*BLI, bioluminescence imaging; FLI, fluorescence Imaging; NI, nuclear imaging; IV, intravenous; IM, intramuscular; IC, intracardiac; RO, retro-orbital injection; DiD and DiR, near-infrared dyes; Gluc, Gaussia luciferase; Rluc, Renilla luciferase; O, available; X, limited*.

Numerous preclinical studies involving molecular imaging modalities, either alone or multiple modalities, are needed to improve our knowledge of EV distribution for better clinical translation of the EV-based therapeutic approaches. Here, we discuss the highlights of using molecular imaging approaches for monitoring EVs in animal models. Furthermore, factors that affect detection sensitivity, such as methods of EV labeling, efficiency of EV labeling, toxicity, and limits of detection of imaging modalities as well as the future prospects of the use of EVs have been discussed.

### Molecular imaging techniques for *in vivo* monitoring of EVs

Optical imaging is a powerful tool for cell tracking in small animals over the desired time periods without sacrificing the subjects (Kim et al., 2015; Li et al., 2016). Optical imaging consists of two main types: (i) Fluorescence protein imaging that involves the use of endogenous or exogenous molecules or materials that emit light when activated by an external light source such as a laser (Figures 3A,B); and (ii) Bioluminescent imaging that involves the use of a natural light-emitting protein that is activated by a chemical reaction, such as luciferase to trace the movement of certain cells or to identify the location of specific chemical reactions within the body (Figures 3A,B).

Nuclear imaging using radionuclides has been widely used for imaging human and mouse models. Radionuclides emit radiation that can be detected in living animals using a specific camera. Nuclear imaging provides excellent sensitivity and good tissue penetration (Ahn, 2014) such that it enables the visualization of deep organs such as the liver and spleen (Gangadaran et al., 2017a). MRI was conventionally used for anatomical imaging with high resolution and good tissue contrasts. The recent advancements in development of MR agents make it applicable to visualize the *in vivo* localization of EVs.

#### Monitoring *in vivo* biodistribution of EVs by fluorescence imaging

Fluorescence imaging involves the use of an external light source and a low-light camera with appropriate filters to collect fluorescence emission lights from samples (Kim et al., 2015). Recent advances in this system allow real-time visualization of EVs in an animal model (Grange et al., 2014; Lai et al., 2015; Smyth et al., 2015). Near-infrared (NIR) dyes are suitable for non-invasive *in vivo* applications because of their high signal-to-noise ratio, low auto fluorescence of biological tissue in the 700–900 nm spectral range, and deep tissue penetration of the NIR light. In particular, DiR (1,1′-Dioctadecyl-3,3,3′,3′-Tetramethylindotricarbocyanine Iodide) is a lipophilic dye, weakly fluorescent in H_2_O but fluorescent and photo-stable when incorporated into lipid membranes (of cells or EVs).

Fluorescent proteins were applied to visualize endogenous and exogenous EVs and track cell-to-cell communication in animal models. However, these proteins show low tissue penetration and this technique does not allow non-invasive *in vivo* imaging because of low resolution and the imaging can be performed only after the animal is sacrificed or surgically exposed (Hoshino et al., 2015; Lai et al., 2015; Zomer et al., 2015). Although fluorescent proteins fused with membrane proteins are extensively expressed in EVs, only a small population of EVs express the fluorescent proteins and the signal intensity was variable, according to the amount of reporter protein expression (Choi and Lee, 2016). Compared to lipophilic dyes, fluorescent reporter protein imaging systems are more specific to EVs. However, these systems require genetically engineered cells, which may change the characteristics of the parent cells and these changes can occur in the EVs as well.

Labeling of EVs by direct fluorescence has been widely used to investigate non-invasive *in vivo* behavior of exogenous EVs in small animal models (Ohno et al., 2013; Grange et al., 2014; Smyth et al., 2015). The labeling process is simple and lipophilic dyes are suitable for real-time monitoring of EVs in their native environments by NIR fluorescence imaging. Lipophilic dyes, including PKH, DiI, DiD, cy7, and DiR, are commonly used and yield stable fluorescent signals *in vitro* as well as *in vivo* (Grange et al., 2014; Watson et al., 2016; Jung et al., 2017).

A recent study used direct labeling of EVs with a dye and reported the biodistribution of breast cancer-derived EVs in mice. EVs derived from different breast cancer cells showed different *in vivo* and *ex vivo* distribution in after 24 h of administration (Wen et al., 2016). Wiklander et al. studied the biodistribution of EVs in mice after a systemic delivery; EVs were isolated from three different murine cell sources, including DCs and labeled with a NIR lipophilic dye. The route-of-administration and the dose and cell source of EVs influenced the biodistribution pattern, as demonstrated by *in vivo* imaging of DiR-labeled EVs (Wiklander et al., 2015). Watson et al. showed the efficient production of engineered EVs from HEK293 cells and labeled them with DiR. Further, live animal imaging showed dramatically lesser liver uptake of EV and increased EVs in blood by pre-treatment with scavenger receptor-A blocker (Watson et al., 2016). Gangadaran et al. recently used an NIR dye (DiR) to study the biodistribution of thyroid cancer (CAL62)-derived EVs in mouse models. Intravenously injected EVs were predominantly distributed to the liver and the spleen followed by the lung and the kidney (Gangadaran et al., 2017b).

Dye-based optical imaging is limited to exogenous EVs and the labeled fluorescent dyes stays in tissues even after EVs are degraded or internalized by the cells. As lipid labeling is non-specific for intact EVs, fluorescence signals can be emitted by cells in which EVs were internalized or attached to cell surface (Wiklander et al., 2015; Choi and Lee, 2016). The major limitation, however, is that the labeling with lipophilic dyes promotes clumping of EVs and may give rise to artifacts, especially during *in vivo* imaging (Grange et al., 2014). Moreover, the repeated washing steps required to remove the free dye residues might end up in significant EV damage. The lipophilic dye remains in the animal tissues even after the clearance of labeled EVs from the system because of several days of estimated *in vivo* half-life of the dye (Lai et al., 2014). In addition, a recent study compared the dye-based direct labeling with the *Renilla* luciferase (Rluc)-based indirect labeling of EVs derived from thyroid cancer cells. This study revealed that labeling with the dye can affect the normal distribution of EVs in an animal model; EV organotropism, which occurs due to the integrins present in the EV membrane, could be influenced by the dye attached onto the EV surface membrane, thus leading to different *in vivo* distribution (Gangadaran et al., 2017b).

#### Monitoring *in vivo* biodistribution of EVs by bioluminescence imaging

Bioluminescence is produced by a chemical reaction between bioluminescent proteins and their appropriate substrates (firefly luciferase and D-luciferin, *Renilla* or *Gaussia* luciferases-coelenterazine) (Wilson and Hastings, 1998; Li et al., 2016; Kalimuthu et al., 2017). Bioluminescence imaging has enabled the real-time visualization of EVs in an *in vivo* animal model and helped study the biodistribution of EVs. Furthermore, bioluminescence imaging offers sensitivity as well as a broad dynamic range for *in vivo* quantification (Takahashi et al., 2013; Imai et al., 2015). Compared to fluorescent-based imaging, bioluminescent imaging has an extremely high signal-to-noise ratio, because the auto-luminescence in mammalian tissue is negligible. Bioluminescence imaging has very low background emission and is independent from an excitation source to emit light. Therefore, bioluminescence imaging has been extensively used to determine cellular distribution, survival, proliferation, and differentiation after transplantation in the development of cell-based therapies (Kim et al., 2015). Takahashi et al. generated a fusion protein be made up of *Gaussia* luciferase (Gluc) and a lactadherin. This genetically engineered EV revealed the spatio-temporal distribution of EVs in a quantifying manner (Takahashi et al., 2013).

Lai et al. combined Gluc and biotinylation to create a EV reporter for multi-modal *in vivo* imaging. They monitored EVs in various major organs and body fluids (blood and urine) after administration of the bioluminescent EVs. Furthermore, they revealed that the EVs first undergo a fast distribution followed by an extended elimination via hepatic and renal routes within 6 h (Lai et al., 2014). Imai et al. used Gluc-lactadherin EVs and revealed that macrophages play significant roles in the clearance of intravenously injected EVs from the circulation (Imai et al., 2015). In another study, the fusion protein of Gluc-lactadherin was used to evaluate the pharmacokinetics of EVs from five different cells (Charoenviriyakul et al., 2017). Recently, Gangadaran et al. generated cancer cells expressing the bioluminescent reporter gene *Rluc* to study the biodistribution in nude mice. EVs were isolated from cancer cells (thyroid cancer: CAL62 and breast cancer: MDA-MB-231) expressing RLUC, and injected intravenously and imaged. CAL62-derived EVs mostly distributed to the lung, followed by the liver, spleen, and kidney, whereas MDA-MB-231-derived EVs distributed to the liver, followed by the lung, kidney, and spleen (Gangadaran et al., 2017b).

However, there are some limitations in using the bioluminescent system. Bioluminescent signal can be reduced when the EVs are located in deep internal organs (Ahn, 2014). Injection of substrates is required to generate optical signals and these substrates might be toxic to the animal and long-term sequential imaging might be technically limited by the multiple injection procedure involved (Gangadaran and Ahn, 2017). Procedures of bioluminescent labeling are complicated, compared with those of fluorescence dyes, because cells undergo a bioluminescent gene transduction, which might modify the natural behavior of the transduced cells (Gangadaran et al., 2017a). In addition, the transduction process is a time consuming.

#### Monitoring *in vivo* biodistribution of EVs by nuclear imaging

Nuclear imaging could be a good option for tracking EVs and evaluating their biodistribution. In this method, three-dimensional images are obtained using single-photon emission computed tomography (SPECT) or positron emission tomography (PET). Furthermore, nuclear imaging combined with anatomical imaging, such as computed tomography (CT) or MRI is also available, and this combined imaging technology provides a better understanding of the localization of the EVs (Figure 4).

Recently, ^111^In-oxine and ^99m^Tc-hexamethylpropyleneamineoxime (HMPAO) were used for tracking EVs and EVMs (Hwang et al., 2015; Smyth et al., 2015). These materials were widely used for direct radionuclide labeling of leukocytes. Indium arranges an uncharged pseudo-octahedral complex with three molecules of 8-hydroxyquinoline (oxine). As this complex is neutral and lipid-soluble, it can penetrate the lipid bilayer of EVs easily and indium becomes decisively attached to cytoplasmic components such as lactoferrin (Roca et al., 2010). Smyth et al. labeled exosomes with ^111^In-oxine and then analyzed their biodistribution (Smyth et al., 2015).

Hwang et al. labeled EVMs with ^99m^Tc-HMPAO and successfully imaged the EVMs using SPECT/CT (Hwang et al., 2015). As ^99m^Tc-HMPAO is a lipophilic agent, it can penetrate the lipid bilayer of EV/EVMs. ^99m^Tc-HMPAO reacts with reducing agents such as glutathione inside the EV/EVMs and is then converted to its hydrophilic form. Therefore, ^99m^Tc could remain inside the EV/EVMs (de Vries et al., 2010). Compared to ^111^In, ^99m^Tc is much cheaper and provides better image quality on gamma camera imaging; however, ^111^In is preferable for delayed imaging owing to its long half-life (2.8 days). ^99m^Tc-HMPAO-labeled EV/EVMs are well-visualized using a gamma camera or SPECT (Hwang et al., 2015; Gangadaran et al., 2017a). As glutathione plays a major role in ^99m^Tc-HMPAO labeling, glutathione concentration of the EVs might be important. Although most cells present glutathione, its concentration varies with the cell type (Gamcsik et al., 2012). The efficiency of labeling EVs with ^99m^Tc-HMPAO might vary with the parent cells of EVs.

Varga et al. used ^99m^Tc-tricarbonyl for labeling EVs. ^99m^Tc-tricarbonyl binds to numerous amino acids, such as histidine, cysteine, and methionine, that might be bound to the surface of EVs (Varga et al., 2016). ^99m^Tc-tricarbonyl showed relatively higher labeling efficiency in RBC-derived EVs (38.8%) with 98% radiochemical purity. However, they only performed 1-h imaging and image-based analysis. Therefore, further studies are needed to validate the efficiency of this labeling method.

Radioiodine (^123^I, ^124^I, ^125^I, and ^131^I) could be an option for labeling EVs (Gangadaran et al., 2017a). ^123^I and ^131^I can be used for gamma camera or SPECT imaging, and ^124^I can be used for PET imaging. Although each radioiodine isotope has different physical properties (such as, half-life, and emitting energy), these isotopes have identical chemical properties; therefore, same labeling methods can be used for these isotopes. Morishita et al. generated exosomes expressing streptavidin-lactadherin fusion protein and then labeled these exosomes with ^125^I-biotin using the streptavidin-biotin system (Morishita et al., 2015). They showed good stability of labeled radioiodine and well-demonstrated the biodistribution of these labeled exosomes. However, image acquisition was not possible because they used ^125^I. In their previous study, they synthesized ^123^I-biotin and performed gamma camera imaging, but the radiochemical yield of ^123^I-biotin is lower than that of ^125^I-biotin (29% vs. 65%) (Kudo et al., 2009). To apply this labeling method to other EVs, the desired gene must be delivered into the target cells and the expression of the protein in EVs must be determined. One big limitation of this transduction technology is the possibility of altering EV characteristics by the transduction procedure. Furthermore, for nuclear imaging, well-trained personnel are needed for the safe handling of the radionuclide. High cost and regulatory policies for the use of radioactive molecules are the other hurdles for using nuclear imaging.

#### Monitoring *in vivo* biodistribution of EVs by MRI

In recent studies, EVs were loaded with MRI contrasts, and the location of these EVs was visualized using MRI. Hu et al. loaded melanoma exosomes with ultra-small super paramagnetic iron oxide (USPIO) (Hu et al., 2014). They loaded EVs with 5 nm-sized nanoparticles, which show low signal intensity in T2-weighted images, by electroporation (54.9 μg iron per 100 μg EV protein). After injection of these EVs into the feet of the mice, MRI successfully revealed the migration of these EVs to the draining lymph nodes. As exosome aggregation or fusion could occur during electroporation, recent studies used a biocompatible trehalose-based electroporation pulse media; using this unique pulse media, melanoma exosomes were loaded with 5 nm USPIO while minimizing the electroporation-induced aggregation effect (Hu et al., 2014). Busato et al. loaded parent cells (adipose stem cells) with USPIO and collected the EVs from these cells (Busato et al., 2016). As they did not perform electroporation or the other manipulation in the EVs, the integrity of the EV membranes could be preserved. However, the amount of USPIO in EVs was much lower than that reported in previous studies (Hood et al., 2014; Busato et al., 2016). For tracking EVs via MRI, a large amount of USPIO-loaded EVs are needed because of the inherent low sensitivity of MRI technology. Only the EVs remaining at the injection sites after intramuscular injection were observed in this study (Busato et al., 2016).

### Tracking EVs and monitoring tumor and metastatic behaviors using molecular imaging

In the last decade, major developments have been made in characterizing the cellular source and role of EVs. The finding that they are natural carriers of miRNA, mRNA, and proteins led to the hypothesis that they can be used as therapeutic agents and vehicles for the delivery of therapeutic cargoes (exogenous) to tumors. Furthermore, the role of EVs in tumor metastasis would help researchers to understand how a tumor prepares the metastatic site at distal organs. Ohno et al. successfully showed that injected exosomes by intravenously delivered let-7a miRNA to an EGFR-expressing tumor by DiR labeling of the EVs in mice and inhibited tumor growth (Ohno et al., 2013). Smyth et al. observed a comparable fast clearance and limited tumor accumulation of injected EVs labeled with DiR and ^111^In-oxine by intravenous route (Smyth et al., 2015). Bellavia et al. engineered HEK293T to express the EV protein Lamp2b, fused to a portion of interleukin 3 (IL3) to target specific cancer cells (chronic myelogenous leukemia); DiR was used to label the HEK293T-derived EVs (Bellavia et al., 2017). Watson et al. showed efficient enhanced tumor delivery of engineered EVs by using DiR-labeled EVs, and living animal imaging showed dramatically reduced liver uptake of EVs and increased EVs circulating in blood; the EVs were eventually targeted to the tumor by pre-treatment with scavenger receptor-A blocker (Watson et al., 2016). Lai et al. combined Gluc and biotinylation to create an EV reporter for multi-modal *in vivo* imaging. They administered bioluminescent EVs and monitored their targeting to the tumor. Furthermore, they revealed that the EVs first undergo a quick distribution followed by targeting to tumor within 60 min (Lai et al., 2014).

Hoshino et al. directly labeled the EVs with PKH67 (green) or PKH26 (red) membrane dye, and the fluorescently labeled EVs were systemically injected (the tail vein, retro-orbital venous sinus, or intracardially) into nude mice. They quantified EV distribution and uptake in organs by NIR and confocal microscopic imaging, and demonstrated that integrins of EVs could be exploited to predict the organ-specific metastasis of tumors (Hoshino et al., 2015). Zomer et al. demonstrated that *in vivo* imaging of EVs revealed metastatic behavior similar to that observed using fluorescent protein with high-resolution intravital imaging on surgically exposed imaging site in a mouse model (Zomer et al., 2015). Hu et al. showed sentinel lymph node using MRI after injection of EVs loaded with USPIO into the foot pad (Hu et al., 2014; Table 3).

**Table 3:** Tumor targeting and tumor metastatic behavior of extracellular vesicles, as assessed by molecular imaging strategies.

**Imaging technique**	**Labeling strategy**	**Labeling molecule**	**EV source**	**Administration route**	**Purpose**	**Subject**	**Clinical translation**	**References**
FLI	Indirect	EGFP, dsRED	Breast cancer	Spontaneous	Metastatic behavior	Mice	X	Smyth et al., 2015
	Direct	PKH67,26	Breast cancer	RO, IV, IC	Organotropic metastasis	Mice	X	Hoshino et al., 2015
		DiR	HEK293	IV	Tumor targeting	Mice	X	Ohno et al., 2013
			4T1, MCF-7, & PC3	IV, IT	Tumor targeting	Mice	X	Smyth et al., 2015
			HEK293T	IP	Tumor targeting	Mice	X	Bellavia et al., 2017
			HEK293	IV	Tumor targeting	Mice	X	Watson et al., 2016
		Cy7	4T1	IV	Tumor targeting	Mice	X	Jung et al., 2017
BLI	Indirect	Gluc	HEK293T	IV	Tumor targeting	Mice	X	Lai et al., 2014
NI	Direct	^111^In-oxine	4T1, MCF-7, & PC3	IV, IT	Tumor targeting	Mice	O	Smyth et al., 2015

*BLI, bioluminescence imaging; FLI, fluorescence Imaging; NI, nuclear imaging; GFP, Green fluorescence protein; RFP, Red fluorescence protein; IV, intravenous; IT, intratumor; IM, intramuscular; IC, intracardiac; RO, retro-orbital injection; DiR, near-infrared dyes; Gluc, Gaussia luciferase; O, available; X, limited*.

### Molecular imaging for monitoring targeted EVs in non-cancerous diseases

EVs are not only used in targeting tumors (Lai et al., 2014; Watson et al., 2016) but also in other diseases that can be monitored by molecular imaging (Table 4; Grange et al., 2014; Gangadaran et al., 2017c). DiD-labeled EVs were derived from MSCs pre-incubated with the DiD dye, and the isolated EVs were directly labeled with the DiD dye. Further, the labeled EVs were found to be accumulated predominately in the mice kidneys with acute kidney injury, and directly labeled EVs showed a prolonged signal in the animal model (Grange et al., 2014). Recently, Gangadaran et al. used fluoresce imaging to monitor EVs derived from MSCs and demonstrated prolonged *in vivo* retention of the EVs by mixing with scaffold in an ischemic hindlimb mouse model (Gangadaran et al., 2017c). Rajendran et al. used a NIR dye (DiR) to determine the treatment interval duration for EVs derived from MSCs at an intradermal site. EVs remained at the injection site (intradermal) for 48 h and were then distributed to the internal organs (lungs, liver, and kidneys) by 72 h; no signals were then observed at the injection site (Rajendran et al., 2017). Lai et al. reported successful visualization of EV-mediated communication between cells by imaging using GFP and tdTomato. Here, they transduced the cell with the fluorescent proteins and then isolated the EVs from the cells; they used multiphoton intravital microscopy to analyze the EV-RNA cargo delivery (Lai et al., 2015).

**Table 4:** Tracking extracellular vesicles for target/delivering to non-cancerous diseases using molecular imaging strategies.

**Imaging technique**	**Labeling strategy**	**Labeling molecule**	**EV source**	**Administration route**	**Purpose**	**Subject**	**Clinical translation**	**References**
FLI	Indirect	EGFP, tdTomato	293T	Skin surface	Delivery EV-RNA cargo	Mice	X	Lai et al., 2015
	Direct	DiD	MSC	IV	Targeting acute kidney injury	Mice	X	Grange et al., 2014
		DiD	MSC	IM	Intramuscular tissue internalization	Mice	X	Gangadaran et al., 2017c
		DiR	MSC	ID	Dermal papilla activation	Mice	X	Rajendran et al., 2017
MRI	Direct	USPIO	B16-F10	Food pad	Lymph nodes	Mice	O	Hu et al., 2014
			Stem cells	IM	Intramuscular internalization	Mice	O	Busato et al., 2016

*FLI, fluorescence Imaging; MRI, magnetic resonance imaging; GFP, Green fluoresce protein; MSC, mesenchymal stem cell; USPIO, ultra-small super paramagnetic iron oxide; IV, intravenous; IM, intramuscular; ID, intradermal; DiD and DiR, near-infrared dyes; O, available; X, limited*.

## Future directions

Development of safe and effective drug delivery systems to desired target sites is receiving increasing attention recently. Number of studies are now shifting their focus from synthetic carriers to biological carriers that can achieve better efficacy and safety (Kim et al., 2017b). EVs have many pathophysiological functions, which might be helpful for drug delivery to and treatment of target lesions. In this aspect, EVs might have superiority over other synthetic carriers.

Bioengineering of parent cells might be helpful for enhancing the favorable characteristics of EVs as drug delivery vehicles. It can increase the targetability of EVs to lesions or enhance their therapeutic effect by loading useful biomaterials (Gujrati et al., 2014; Kim et al., 2017a). However, these bioengineering methods could be another hurdle for clinical translation.

EVs can be labeled or loaded with therapeutic radionuclides such as alpha-emitting (e.g., ^211^At, ^213^Bi) or beta-emitting (e.g., ^131^I, ^90^Y, ^177^Lu) radionuclides, which are widely used for radionuclide therapy (Ahn, 2014). As USPIOs can mediate magnetic hyperthermia, EVs may be used as theranostic nanocarriers to simultaneously detect and treat tumor microenvironments (Hood, 2016).

As drugs loaded into EVs show different pharmacokinetics, we can find new application of withdrawn drugs, which have good therapeutic effects but fail to reach the target tissue or have adverse effects in non-target tissues. Furthermore, insoluble drug candidates could be loaded into the EVs and effectively delivered to target tissues.

However, various components and low production yield of EVs are obstacles for clinical translation. EVMs derived from cells could be a good solution for the low production yield of EVs (Jang et al., 2013). EVMs could be a good substitute for EVs as drug delivery vehicles. Both EVs and EVMs have complex components, which need to be assessed for toxicity. To overcome this issue, self-derived EVs and EVMs from patients can be used (Escudier et al., 2005; Morse et al., 2005; Besse et al., 2016).

Several different labels and imaging techniques have been explored for labeling and tracking EVs in animal models to understand cancer biology and to develop EV-based targeted therapies. Direct labeling of EVs with lipophilic dye is easier than the indirect labeling methods and the safety profiles of direct labeling with dyes are relatively better than those of indirect labeling. However, the disadvantage of direct cell labeling is that the dye itself is detected rather than the EVs of interest (Choi and Lee, 2016). Although there are many reporter luciferase gene-based cell labeling studies available (Kim et al., 2015), unfortunately the luciferase reporter gene-based EV labeling studies are very few (Gangadaran et al., 2017a). Although luciferases can enable *in vivo* long-term monitoring of the cells in a quantitative manner in small animal models (Li et al., 2016), such long-term monitoring cannot be performed for EVs (Lai et al., 2014). Therefore, care must be taken when interpreting the experimental results, and rigorous validation is certainly needed to obtain more robust and reliable data.

Although there are some obstacles to use radionuclides in experiments, nuclear imaging has major advantage of no depth limitation compared to the optical imaging. After injection, most of the EVs are visualized in the liver and spleen; therefore, optical imaging is not sufficient for tracking EVs. Nuclear imaging can provide three-dimensional images by SPECT or PET, and the combined use of nuclear imaging and CT or MRI enhances the anatomical localization of EVs. Nuclear imaging can also provide semi-quantitative parameters. To the best of our knowledge, no literature data are available for the *in vivo* PET imaging of EVs; however, indirect labeling of EVs with positron-emitting radionuclides will be developed for the best nuclear *in vivo* imaging of EVs (Ahn, 2014).

Although MRI contrast agents have shown relatively lower sensitivity than that of optical imaging or nuclear imaging (Ahn, 2014), technological advances might provide better imaging contrast agents and machines. Nowadays, promising novel imaging sequences, such as SWIFT and UTE, provide positive contrast for SPIO, and it could be possible to characterize the distribution of exosomes using MRI (Hu et al., 2014).

Safety of labeled EVs is always an immense concern in potential clinical studies, as the introduction of foreign materials such as dye, radionucleotides, magnetic parties, and reporter proteins may cause unpredictable results in patients. As radionuclides are already used in clinics, there is less ethical and legal obstacles in clinical translation compared to those observed when using the other new probes. Recently, some clinical trials were performed to treat advanced melanoma and lung cancer by EVs (Escudier et al., 2005; Morse et al., 2005; Besse et al., 2016); however, there is no report on the kinetics of EVs in the human body. Molecular imaging techniques should be helpful to elucidate the *in vivo* kinetics of EVs used as drug delivery vehicles in humans.

## Conclusion

In recent years, EVs have become an enthusiastic subject as drug delivery vehicles. Nonetheless, action mechanism, biodistribution, and pharmacokinetics of exogenously administered EVs are not well-studied, and the possibility of targeted delivery of drugs using EVs has not been fully assessed. *In vivo* molecular imaging of EVs would contribute to the understanding of the pathophysiological influence of EVs and accelerate the development of EV therapeutics in clinical fields. Application of optimal molecular imaging technology is needed to ensure the efficient use of EVs in various specific study situations.

## Author contributions

PG, CH, and B-CA: Contributed to the conception, writing, and discussion of this review; PG and CH: Wrote the initial draft of the manuscript. The final version was approved by all the authors.

### Conflict of interest statement

The authors declare that the research was conducted in the absence of any commercial or financial relationships that could be construed as a potential conflict of interest. The reviewer DL and handling Editor declared their shared affiliation.

## Abbreviations

CT: computed tomography
DCs: dendritic cells
ESCRT: endosomal sorting complex required for transport
EVM: extracellular vesicle mimetics
EVs: extracellular vesicles
HMPAO: ^99m^Tc-hexamethylpropyleneamineoxime
MRI: magnetic resonance imaging
MSC: mesenchymal stem cell
NIR: Near-infrared
PDCD6IP: programmed cell death 6 interacting protein
PET: positron emission tomography
SPECT: single-photon emission computed tomography
TSG101: tumor susceptibility gene 101
USPIO: ultra-small super paramagnetic iron oxide.
